# SARS-CoV-2 infection of sustentacular cells disrupts olfactory signaling pathways

**DOI:** 10.1172/jci.insight.160277

**Published:** 2022-12-22

**Authors:** Abhishek Kumar Verma, Jian Zheng, David K. Meyerholz, Stanley Perlman

**Affiliations:** 1 Department of Microbiology and Immunology and; 2 Department of Pathology, University of Iowa, Iowa City, Iowa, USA.

**Keywords:** COVID-19, Mouse models

## Abstract

Loss of olfactory function has been commonly reported in SARS-CoV-2 infections. Recovery from anosmia is not well understood. Previous studies showed that sustentacular cells, and occasionally olfactory sensory neurons (OSNs) in the olfactory epithelium (OE), are infected in SARS-CoV-2–infected patients and experimental animals. Here, we show that SARS-CoV-2 infection of sustentacular cells induces inflammation characterized by infiltration of myeloid cells to the olfactory epithelium and variably increased expression of proinflammatory cytokines. We observed widespread damage to, and loss of cilia on, OSNs, accompanied by downregulation of olfactory receptors and signal transduction molecules involved in olfaction. A consequence of OSN dysfunction was a reduction in the number of neurons in the olfactory bulb expressing tyrosine hydroxylase, consistent with reduced synaptic input. Resolution of the infection, inflammation, and olfactory dysfunction occurred over 3–4 weeks following infection in most but not all animals. We also observed similar patterns of OE infection and anosmia/hyposmia in mice infected with other human coronaviruses such as SARS-CoV and MERS-CoV. Together, these results define the downstream effects of sustentacular cell infection and provide insight into olfactory dysfunction in COVID-19–associated anosmia.

## Introduction

The global outbreak of COVID-19, caused by SARS-CoV-2, has resulted in infection of over 613 million people and 6.5 million deaths worldwide, as of September 21, 2022 (https://covid19.who.int/). SARS-CoV-2 is a respiratory coronavirus that targets the upper and lower respiratory tracts. The symptoms and signs of disease include mild-to-medium fever, cough, diarrhea, fatigue, and dyspnea, culminating in severe cases with acute respiratory distress syndrome (ARDS) (1–4). In addition to pulmonary and systemic disease, patients have also reported neurological complications including headache, dizziness, ageusia/hypogeusia, anosmia/hyposmia, myalgia, ataxia, and seizures (5–8). In particular, gustatory and olfactory dysfunction (OD) are 2 prominent and hallmark symptoms in SARS-CoV-2–infected patients (9, 10) and is considered a diagnostic criterion for COVID-19 (10–12). The occurrence of respiratory virus–induced alteration of smell has been occasionally identified in patients infected with rhinoviruses, common cold coronaviruses, parainfluenza viruses, and Epstein-Barr virus (13, 14), but it is much less common than in COVID-19 patients and most often results from nasal passage obstruction.

We and others have previously reported that sustentacular cells are the major sites of SARS-CoV-2 infection in the olfactory epithelium (OE) (15–19). Several single-cell RNA-Seq studies showed that the SARS-CoV-2 receptor angiotensin-converting enzyme 2 (ACE2) and a protease critical for virus entry, TMPRSS2, are present on sustentacular cells, which act as supporting cells for olfactory sensory neurons (OSNs) in the OE (20, 21). However, the OD associated with SARS-CoV-2 infection cannot easily be explained by sustentacular cell infection alone. During the process of olfaction, inhalation of odorants is followed by binding to the odorant receptor (OR) on OSN cilia. The binding of odorants to the receptor initiates a cascade of signal transduction events involving adenylyl cyclase III (ACIII), leading to the depolarization of neurons (22). The effect of SARS-CoV-2 infection on OSNs and signaling transduction is not well understood because studies show that OSNs do not express ACE2 (20, 21) and that most demonstrate infrequent infection of OSNs, at best (16, 17, 19, 23).

One important aspect of SARS-CoV-2–induced anosmia that is not well defined is the duration of OD after infection. Recovery of the senses of smell and taste has been assessed primarily through self-reporting by patients, which is not quantitative. Reports suggest that anosmia/hyposmia resolve within 2–6 weeks of symptom onset, although OD persists in a small but significant percentage of patients (24, 25). Notably, recovery of olfaction appears to occur more rapidly in COVID-19 patients with hyposmia rather than anosmia (26, 27). A study using the University of Pennsylvania Smell Identification Test (UPSIT), a 40-odorant psychophysical smell test, described nearly full recovery from hyposmia in the majority of patients but also found that patients still scored lower than age- and sex-matched healthy controls for as long as 4–6 months (28). Notably, a few patients remained hyposmic 1 year after disease onset (29, 30). Together, these results suggest that, while most COVID-19 patients with anosmia or ageusia recover nearly completely, there is a subset with prolonged and potentially permanent OD.

Anosmia/ageusia has been identified in several experimental animal models of COVID-19 (15, 17, 18). Original strains of SARS-CoV-2 do not naturally infect mice due to incompatibility between the viral surface (S) glycoprotein and mouse ACE2 (mACE2). To address this incompatibility, we and others developed transgenic mice that expressed human ACE2 (hACE2) following the 2002–2004 SARS epidemic (31–34). K18-hACE2 mice express hACE2 under the cytokeratin 18 (KRT18, called K18 hereafter) promoter, predominantly in epithelial cells, although some cerebral and cerebellar neurons also show hACE2 expression (35). Here, for many of our experiments, we infected K18-hACE2 mice with the 2019n-CoV/USA-WA1/2019 strain of SARS-CoV-2 (SARS-CoV-2 herein).

Alternatively, genetic modification of the spike protein (S) of SARS-CoV-2 using reverse genetics resulted in enhanced binding to mACE2 (2, 36–38). Original strains of SARS-CoV-2 were adapted for mouse infection by targeting amino acids at positions 498/499 (Q498Y/P499T) or 501 (N501Y). We engineered a virus that expressed N501Y and demonstrated that this mouse-adapted virus caused minimal disease in mice (37). However, after serial passage through mouse lungs, we isolated a virus, SARS2-N501Y_MA30_, that causes severe pulmonary disease in unmanipulated laboratory mice.

Using these 2 mouse models of SARS-CoV-2 infection, we performed a temporal analysis of anosmia from infection onset to resolution of clinical disease. The results indicate that the early phase of infection was accompanied by anosmia/hyposmia and was characterized by extensive cilia damage and downregulation of OR expression. As the infection resolved, anosmia largely resolved, although olfactory receptor expression did not completely return to levels observed in the absence of infection. Together, these results suggest that sustentacular cell infection has important effects on OSN function mediated in part by reduction in OR levels, providing insight into anosmia in patients.

## Results

### Induction and resolution of anosmia.

**Figure 1:**
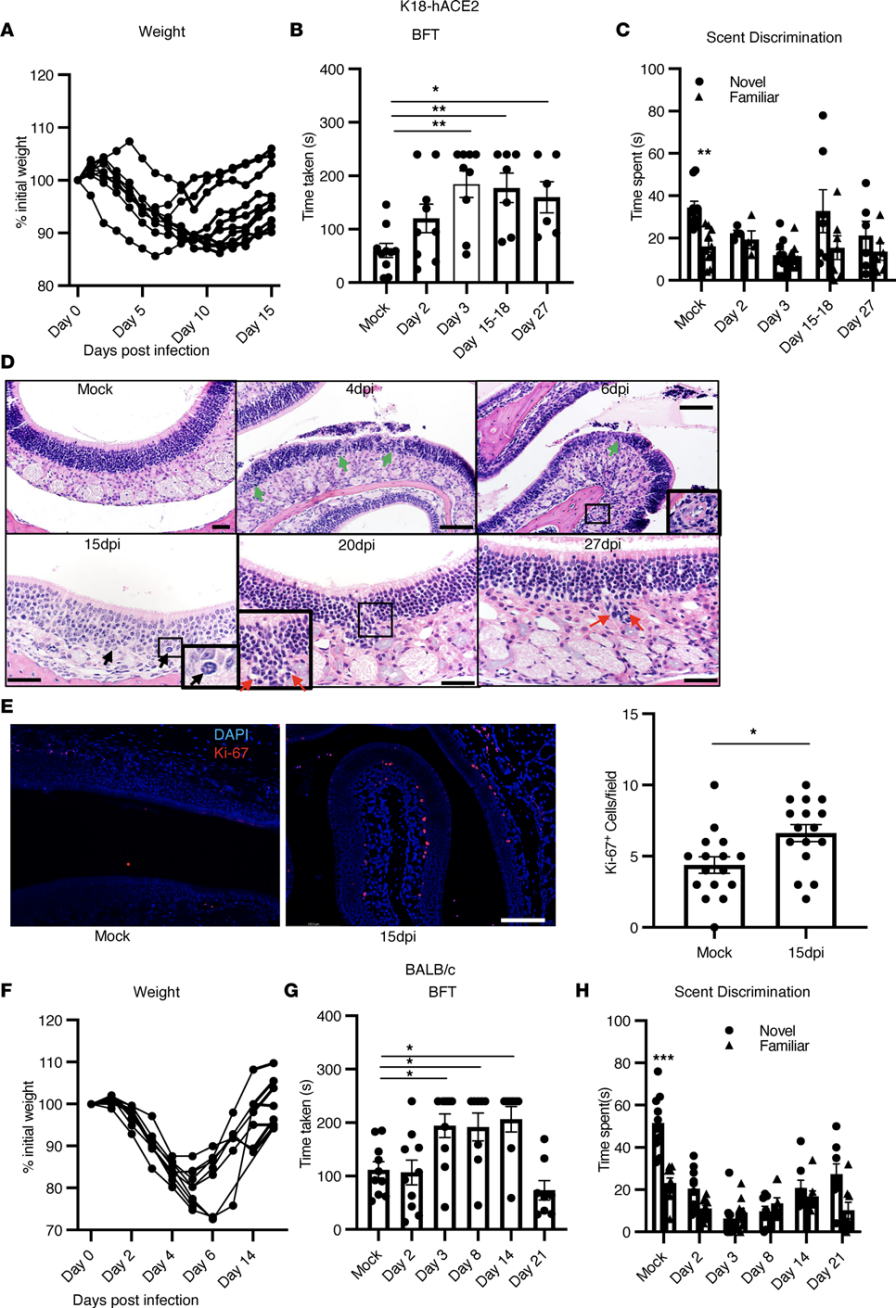
Olfactory dysfunction in SARS-CoV-2–infected mice. (**A**–**E**) K18-hACE2 mice were infected with 2000 pfu SARS-CoV-2 intranasally. (**A**). Weights were monitored. (**B** and **C**) BFT and scent-discrimination tests were performed; *n* = 8 mice. (**B**) Time taken to find hidden food is shown. Data represent mean ± SEM of results pooled from 2 independent experiments; mock (9 mice), day 2 and 3 (9 mice), day 15–18 (7 mice), day 27 (6 mice). Data were analyzed using 1-way ANOVA. *P < 0.05, **P < 0.01. Scale bar: 40 μm. (**C**) Time spent exploring novel and familiar scents. Data represent mean ± SEM of results pooled from 2 independent experiments with 5–10 mice per group. Data were analyzed using 2-way ANOVA. *P < 0.05, **P < 0.01. (**D**) Pathological analysis of OE shows degenerative (green arrow at 4 dpi and 6 dpi) and necrotic changes (inset, 6 dpi). Mitotic cells (black arrow) were observed at 15 dpi in the OE. A few sites of OE disruption and increased cellularity were detected at 20 and 27 dpi (red arrows). (**E**) Ki-67 staining (red) in OE shows increased proliferation in SARS-CoV-2–infected samples. Summary data represent numbers of Ki-67^+^ cells in uninfected and 15 dpi OE per 20× field. Four fields from 4 mice were analyzed using Mann-Whitney *U* tests. *P < 0.05. Scale bar: 50 μm. (**F**–**H**) BALB/c mice were infected with SARS2-N501Y_MA30_. (**F**) Weights were monitored; *n* = 8 mice. (**G** and **H**) BFT and scent-discrimination tests are shown. Data represent the mean ± SEM of results pooled from 2 independent experiments with mock, day 2, and day 3 (10 mice) and days 8, 14, and 21 (8 mice). Data were analyzed using 1-way (**B** and **G**) and 2-way (**C** and **H**) ANOVA. *P < 0.05, ***P < 0.001.

We previously showed that anosmia developed in all SARS-CoV-2–infected K18-hACE2 mice (15). Next, we investigated the recovery phase of anosmia after SARS-CoV-2 infection using buried food tests (BFT) and scent-discrimination tests as described previously (15). During a BFT, a breakfast cereal (Froot Loops; Kellogg Cereals) was hidden in the bedding. Mice were allowed to find food, and the time taken to do so was recorded. Male and female K18-hACE2 mice at 2 and 3 days postinfection (dpi) had minimal weight loss (Figure 1A) and normal mobility, but they were unable to find the buried food or discriminate between novel and familiar scents as compared with uninfected mice (Figure 1, B and C). At 15–18 dpi, these mice were still not able to find food in 4 minutes (the designated observation time) (Figure 1B), while at 27 dpi, approximately 30% of mice still were unable to find the buried food. Similar results were obtained in scent-discrimination tests (Figure 1C). To investigate this further, we examined pathological changes in the olfactory epithelia at 4 dpi, 6 dpi, 15–18 dpi, and 27 dpi days after infection. Localized pathological changes, including degenerative and necrotic changes in the OE, were observed at early times after infection (4 dpi and 6 dpi) (Figure 1D). By 15 dpi, the OE appeared mostly recovered, with rare sites of degeneration, scattered apoptotic cellular debris, and mitotic figures (15 dpi) in the OE (Figure 1D). We confirmed this observation by analyzing cells for expression of a proliferation marker, Ki-67, and observed a greater number of Ki-67^+^ cells in 15 dpi samples as compared with uninfected samples (Figure 1E). However, uncommon focal sites of OE disruption were still detected at this time point, with increased cellularity extending into the lamina propria (20 dpi). By 27 dpi, the OE showed nearly complete recovery from infection on pathological examination. Collectively, these data demonstrate functional as well as anatomical recovery in most mice following SARS-CoV-2–induced anosmia.

Although all mice developed anosmia, a caveat of these experiments is that brain infection occurs in a fraction of infected K18-hACE2 mice (15). Therefore, to eliminate any confounding effects resulting from infection of the brain, we next used a second murine model of SARS-CoV-2 infection. BALB/c mice infected with SARS2-N501Y_MA30_ of all ages and aged C57BL/6 mice do not develop infection of the brain or significant neuropathology but, rather, succumb to respiratory infection after exposure to lethal doses of virus (37). We infected BALB/c mice with a sublethal dose of SARS2-N501Y_MA30_ (Figure 1, F–H) and found that they developed olfactory defects similar to those observed in infected K18-hACE2 mice (Figure 1, G and H). Infected mice developed hyposmia/anosmia at 2 dpi and 3 dpi, with recovery observed between 14 dpi and 21 dpi (Figure 1, G and H). Pathological analyses of OE at several time points after infection showed tissue damage at early times, with repair mostly complete by 15 dpi. At 15 dpi, there were uncommon sites of basal cell hyperplasia characterized by layers of plump nuclei along the basal OE border, which extended into the OE layer replacing hyperchromatic small OE neurons (Supplemental Figure 1A; supplemental material available online with this article; https://doi.org/10.1172/jci.insight.160277DS1). At 21 dpi, the OE appeared largely normal, although a few hyperchromatic OSN nuclei were still present, as were occasional fields of OE that were composed mostly of regenerative epithelia with larger nuclei and the absence of neurons (Supplemental Figure 1A). These data show that the temporal recovery from anosmia is parallel by resolution of pathological changes in the OE resulting from infection.

### Infection of sustentacular cells.

**Figure 2:**
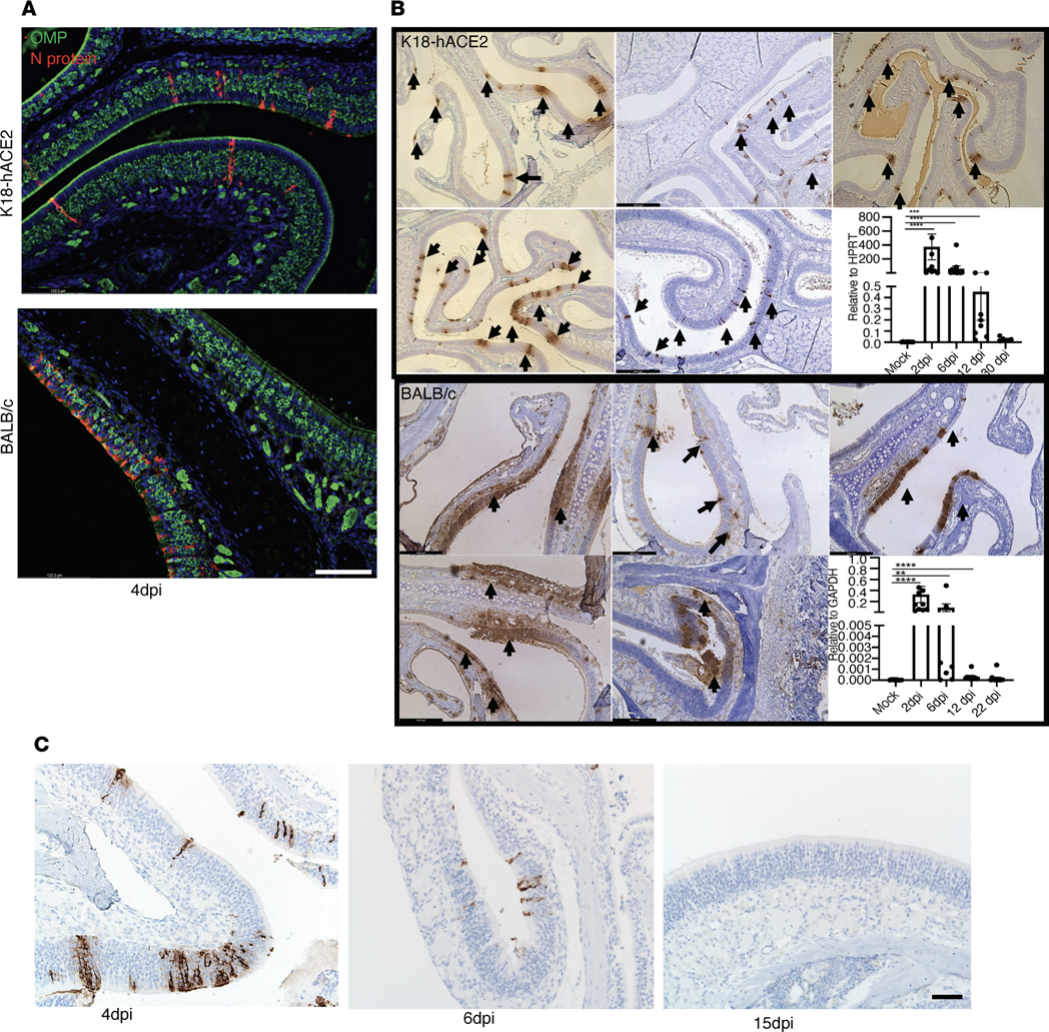
Comparison of sustentacular cell infection in K18-hACE2 and BALB/c mice. (**A**) Sustentacular cells and not OSNs are infected in K18-hACE2 and BALB/c mice. OE sections were prepared and stained with antibodies to SARS-CoV-2–N protein (red) and OMP (green) at 4 dpi. Scale bar: 50 μm. (**B**) Infected cells in K18-hACE2 and BALB/c mice were detected by staining for N protein. Sites of infection are indicated by arrows. Representative images from 5 different mice are shown. Levels of viral RNA measured by PCR at different times after infection are shown in the lower right panels. Data represent mean ± SEM of results pooled from 2 independent experiments with mock (8 mice), 2 and 6 dpi (10 mice), 12 dpi (8 mice), and 30 dpi (6 mice) for K18-hACE2 mice. Mock (8 mice), 2 and 6 dpi (9 mice), and 12 and 22 dpi (8 mice) for BALB/c mice. (**C**) N protein staining was performed in infected K18-hACE2 mice at the indicated days. Scale bar: 50 μm. Data in **B** were analyzed using Mann-Whitney *U* tests. **P < 0.01, ****P < 0.0001.

Previous studies of the OE isolated from humans at autopsy or from experimentally infected animals, including K18-hACE2 mice, showed that sustentacular cells but not OSNs were infected with SARS-CoV-2 (15, 16, 19). We confirmed these results in infected K18-hACE2 mice and extended them to SARS2-N501Y_MA30_–infected BALB/c mice (Figure 2A). Most notably, the pattern of sustentacular cell infection differed when the 2 infections were compared (Figure 2B). Sustentacular cell infection appeared sporadic in K18-hACE2 mice, while it was localized, but it appeared extensive in SARS2-N501Y_MA30_–infected mice. These different patterns of infection may be attributed to differences in mouse strain (BALB/c versus C57BL/6; background of K18-hACE2 mice), differences in virus (WT versus N501Y_MA30_) or perhaps ectopic hACE2 expression. The K18 promoter is active in sustentacular cells (19, 39). In general, the transgenic K18 promoter is active in the same cells as the natural cytokeratin 18 promoter, but there is evidence of some degree of ectopic expression since it is also active in neurons, which do not normally express ACE2 (39). We further investigated the kinetics of virus clearance by IHC using an antibody directed against the nucleocapsid (N) protein of SARS-CoV-2. Infected cells were readily detected at 4 dpi and 6 dpi but not at 15 dpi in both SARS-CoV-2–infected K18-hACE2 and SARS2-N501Y_MA30_–infected BALB/c mice (Figure 2C). These data were confirmed using quantitative PCR (qPCR) (Figure 2B) and RNAScope (Supplemental Figure 1B).

### Olfactory cilia damage after SARS-CoV-2 infection.

**Figure 3:**
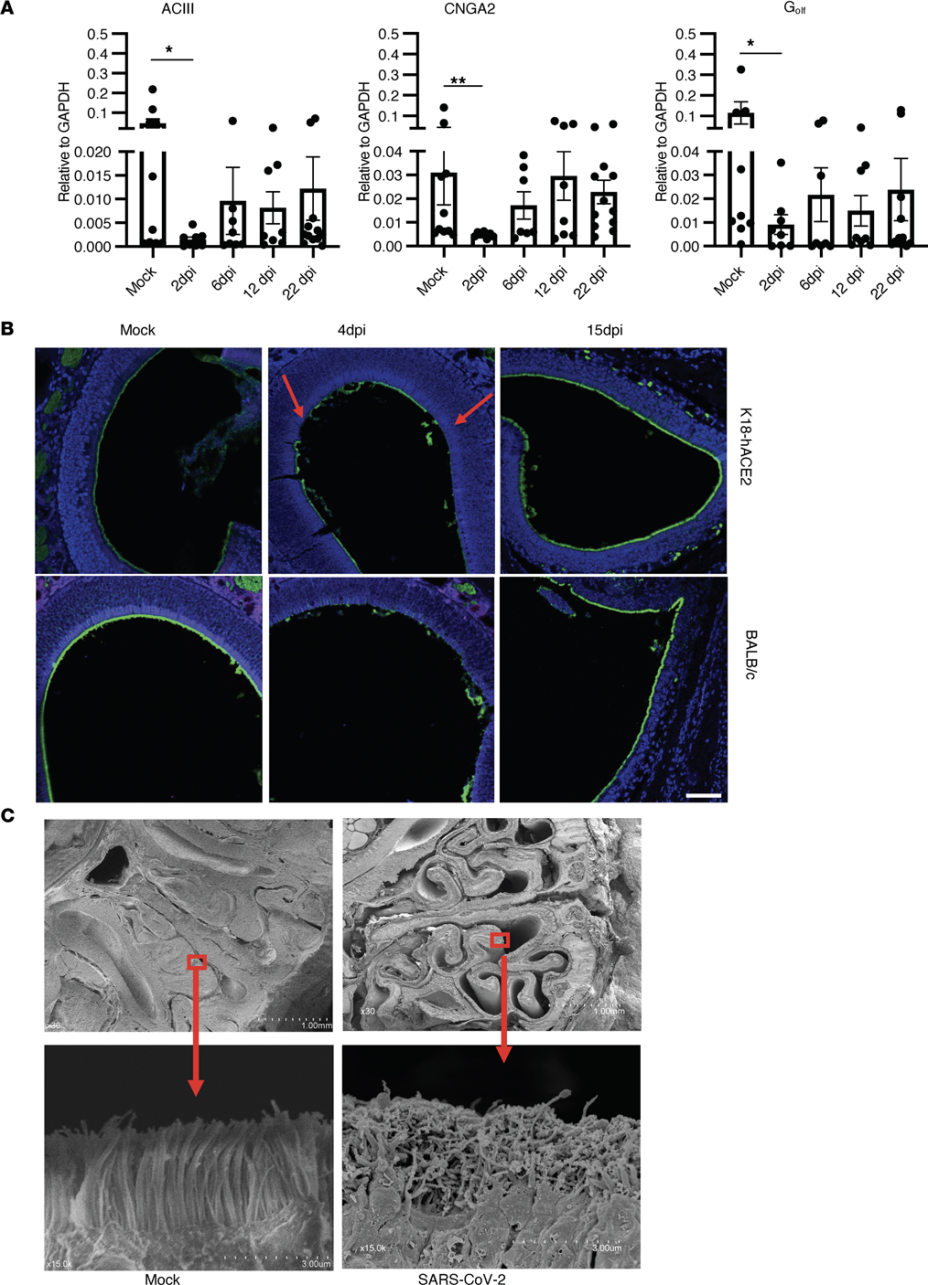
SARS-CoV-2 infection leads to altered signal transduction gene expression and cilia damage in OE. (**A**) Bar graph shows expression profile of signal transduction molecules in SARS2-N501Y_MA30_–infected OE at different time analyzed by qPCR. Data show significantly decreased expression of ACIII, Cnga2, and G_olf_ at 2 dpi. Data represent mean ± SEM of results pooled from 2 independent experiments with mock (10 mice); 2, 6, and 12 dpi (8 mice); and 22 dpi (12 mice). Data were analyzed using Mann-Whitney *U* tests. *P < 0.05, **P < 0.01. (**B**) Cilia in OE were examined by staining for acetylated β-tubulin (green). Arrow marks damaged cillia. Scale bar: 50 μm. (**C**) SEM analysis shows well-aligned lawn of cilia in mock-infected OE but damaged cilia at 4 dpi. Scale bar: 1 mm (upper panels) and 3 μm (lower panels). Arrow shows enlarged view of smaller bracketed area. Scale bar was generated during data acquisition. (**B** and **C**) Data are representative of 4 mice.

These results demonstrate that sustentacular cells were infected but do not provide an explanation for the associated anosmia, since OSNs were not infected. Next, we investigated signal transduction involved in olfaction. Odorant molecules interact with ORs, triggering an increase in the intracellular concentration of cyclic adenosine monophosphate (cAMP) through the activation of receptor-coupled G-protein (G_olf_) and adenylyl cyclase (AC) within cilia. Cyclic nucleotide–gated (CNG) channels located in the ciliary membrane are directly activated by cytoplasmic cAMP, causing an influx of Na^+^ and Ca^+^ ions and, hence, depolarization of OSNs (22, 40–42). All of these components are necessary for olfactory signal transduction and are enriched in olfactory cilia. Alteration in the localization of these components in cilia causes impaired olfactory function (43, 44). First, we examined mRNA expression of *Gnal*, *Adcy3*, and *Cnga2*, as these are molecules involved in olfactory signaling, and we observed decreased mRNA expression of *Gnal*, *Adcy3*, and *Cnga2* in SARS2-N501Y_MA30_–infected BALB/c mice (Figure 3A). Although *Cnga2* expression recovered after 6 dpi, the expression of ACIII and G_olf_ remained low even at 22 dpi. Since the process of transduction starts in the cilia, and decreased expression of transduction molecules was observed, we next investigated whether infection with SARS-CoV-2 had any effect on cilia structure. Staining of OE with acetylated β-tubulin (Ac–β-tubulin) showed well-aligned cilia in uninfected mice, whereas cilia appeared damaged and fragmented in K18-hACE2 and BALB/c mice at 4 dpi. Full recovery was observed by 15 dpi (Figure 3B). Additionally, we examined the topography of olfactory cilia using scanning electron microscopy. Cilia from mock-infected samples displayed a homogeneous columnar appearance, whereas those examined at 4 dpi exhibited irregular shapes and sizes (Figure 3C and Supplemental Figure 1C). Since olfactory receptors are located on cilia and cilia were damaged after SARS-CoV-2 infection, we next examined OR expression.

### OR downregulation after SARS-CoV-2 infection.

**Figure 4:**
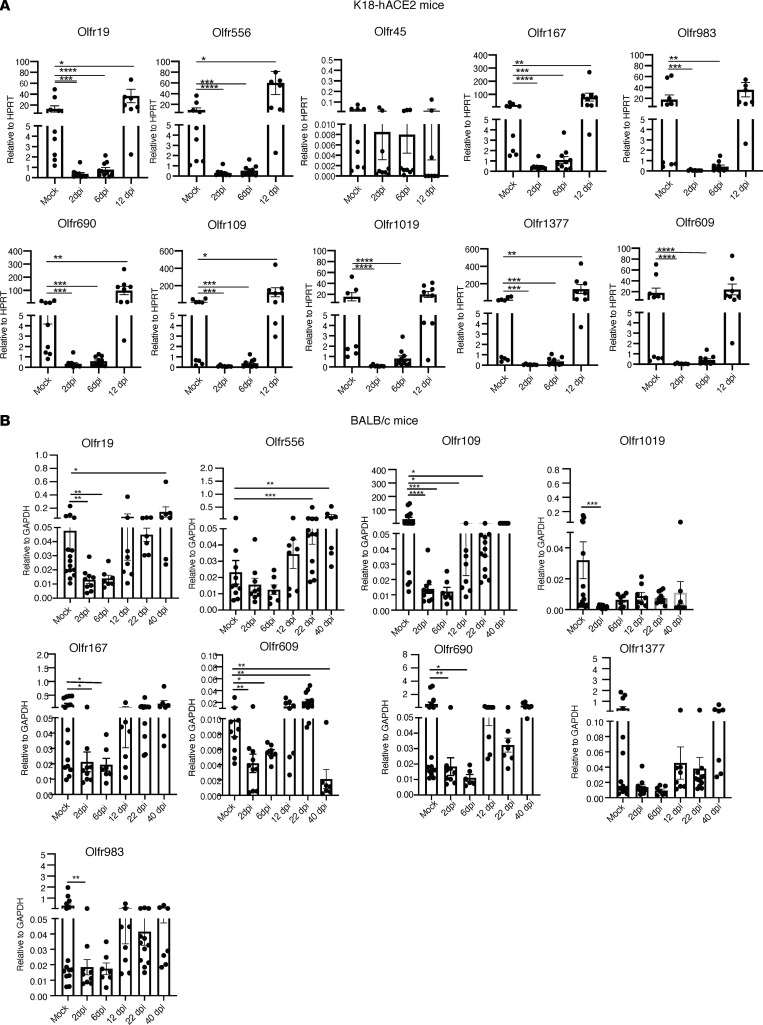
SARS-CoV-2 infection results in altered OR gene expression. OR mRNA expression in SARS-CoV-2–infected K18-hACE2 and SARS2-N501Y_MA30_–infected BALB/c mice were analyzed using qPCR. Data represent mean ± SEM of results pooled from 2 independent experiments. (**A**) K18-hACE2 mice: mock (9 mice); 2, 6, and 12 (10 mice); and 12 dpi (9 mice). (**B**) BALB/c mice: mock (13 mice); 2, 6, and 12 dpi (10 mice); 22 dpi (11 mice); and 40 dpi (7 mice). Data were analyzed using Mann-Whitney *U* tests. *P < 0.05, **P < 0.01, ***P < 0.001, ****P < 0.0001.

OR are evolutionary conserved and essential for olfactory signal transduction. More than 1,000 ORs are expressed in the murine OE, with different patterns of localization. OSN expression of ORs is monogenic and monoallelic so that only a single individual OR is expressed per cell. Next, we analyzed the OE for changes in OR mRNA expression. To examine OR mRNA levels, OE was collected at 2, 6, and 12 dpi from infected K18-hACE2 mice and BALB/c mice. We analyzed the expression of 10 different ORs, partly chosen based on expression in different OE zones (45). In agreement with a previous study of SARS-CoV-2–infected hamsters (46), all OR mRNA levels were significantly decreased at 2 and 6 dpi in K18-hACE2 mice, with virtually full recovery by 12 dpi (Figure 4). Notably, for some OR, mRNA levels were higher at 12 dpi than in mock-infected mice.

Sustentacular cell infection was more focal in SARS2-N501Y_MA30_–infected BALB/c mice (Figure 2B). Consistent with this focal distribution, expression of OR mRNAs were quite variable, with expression of some ORs — such as *Olfr19*, *Olfr109*, *Olfr609*, and *Olfr690* — showing downregulation at 2 and 6 dpi, while expression of others (e.g., *Olfr556*, *Olfr1377*) appeared to be slightly downregulated, but these changes were not statistically significant (Figure 4). As in K18-hACE2 mice, recovery was not uniform. In most cases, levels of OR mRNA were normalized by 12 dpi. On the other hand, levels of some ORs were greater at 40 dpi than in mock-infected mice (e.g., *Olfr19*, *Olfr556*), while they were decreased in 1 OR (*Olfr609*). Together, these data show that OR mRNA expression was often increased after virus clearance in both infected K18-hACE2 and BALB/c mice. This incomplete return to normal levels of OR may result in dysfunctional information processing in primary connections of OSNs.

### Effects on tyrosine hydroxylase expression in the olfactory bulb.

**Figure 5:**
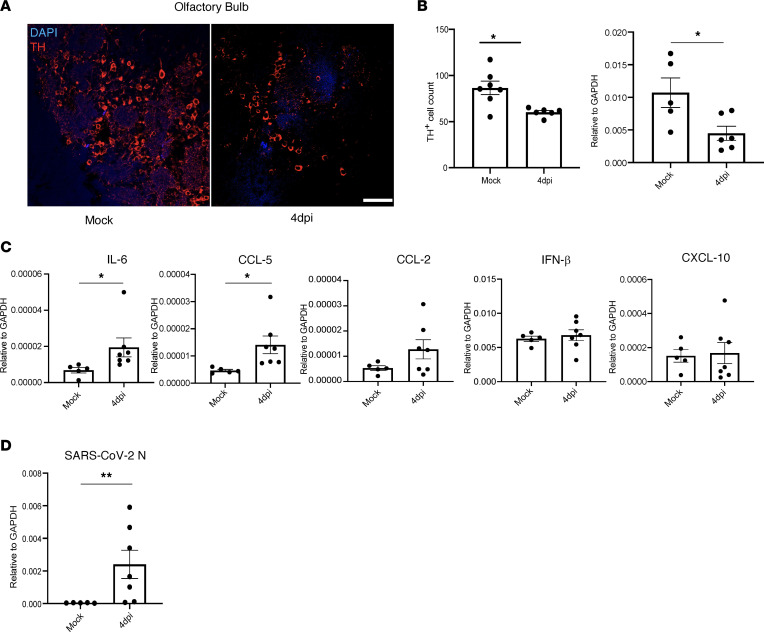
Fewer cells in the OB express tyrosine hydroxylase after SARS2-N501Y_MA30_ infection. (**A**) BALB/c mice were infected with SARS2-N501Y_MA30_. Brains were removed at day 4 p.i., and OB was analyzed for tyrosine hydroxylase (TH) by immunofluorescence staining. (**B**) Quantification of TH^+^ cell numbers/field in mock and infected mice. Numbers of TH^+^ cells in total glomeruli from 3 sections were counted. Average numbers were obtained for each mouse and are shown in the figure. Data represent mock (7 mice) and 4 dpi (6 mice). Each data point represents 1 mouse. Expression of TH mRNA was determined using qPCR. Data represent mean ± SEM were analyzed using Mann-Whitney *U* tests. *P < 0.05. Scale bar: 50 μm. (**C**) mRNA expression of IL-6, CCL-5, CCL-2, IFN-β, and CXCL10 were determined in OB using qPCR. Data represent mean ± SEM of mock (5 mice) and 4 dpi (7 mice). Data were analyzed using Mann-Whitney *U* tests. *P < 0.05. (**D**) SARS-CoV-2–N mRNA expression was analyzed using qPCR from 4 dpi OB. Data represent mean ± SEM of mock (5 mice) and 4 dpi (7 mice). Data were analyzed using Mann-Whitney *U* tests. **P < 0.01.

Next, to examine downstream effects of putative OSN dysfunction, we analyzed the glomerular layer (GL) of the olfactory bulb (OB), since it receives synaptic input from OSN axons. The GL in the OB has an abundant population of periglomerular (PG) neurons surrounding well-demarcated glomeruli. These PG neurons are dopaminergic neurons expressing tyrosine hydroxylase (TH), and their survival is dependent on sensory input from OSNs (47, 48). We investigated TH expression in the OB to indirectly investigate the activity of OSNs. We used SARS2-N501Y_MA30_–infected BALB/c mice for these assays to ensure that the brain infection that sometimes occurs in K18-hACE2 mice did not confound the results. As shown in Figure 5, we readily detected TH expression in the OB, but we also observed a significant decrease in the number of TH^+^ cells in 4 dpi compared with mock-infected samples (Figure 5, A and B). These results were confirmed using qPCR analyses of TH mRNA expression (Figure 5B). OSNs converge at the glomeruli of the OB, which serve as the primary sites of olfactory information processing in the CNS. We investigated if SARS-CoV-2 infection of the OE resulted in an inflammatory response downstream, in the OB. We observed increased levels of IL-6 and CCL5, whereas those of other molecules, such as IFN-β, CCL2, and CXCL10, were not changed when compared with mock-infected samples (Figure 5C and Supplemental Figure 2). Given that we detected changes in some proinflammatory molecules but no infectious virus, we next examined whether there was any viral RNA or protein in the OB. We found no evidence for viral protein by immunofluorescence assays or viral RNA by RNAScope. However, we detected viral RNA using qPCR (Figure 5D). The significance of this finding is uncertain since infectious virus was never detected in the brain, but it may contribute to proinflammatory molecule upregulation.

### Altered myeloid cell distribution and cytokine expression after SARS-CoV-2 infection.

**Figure 6:**
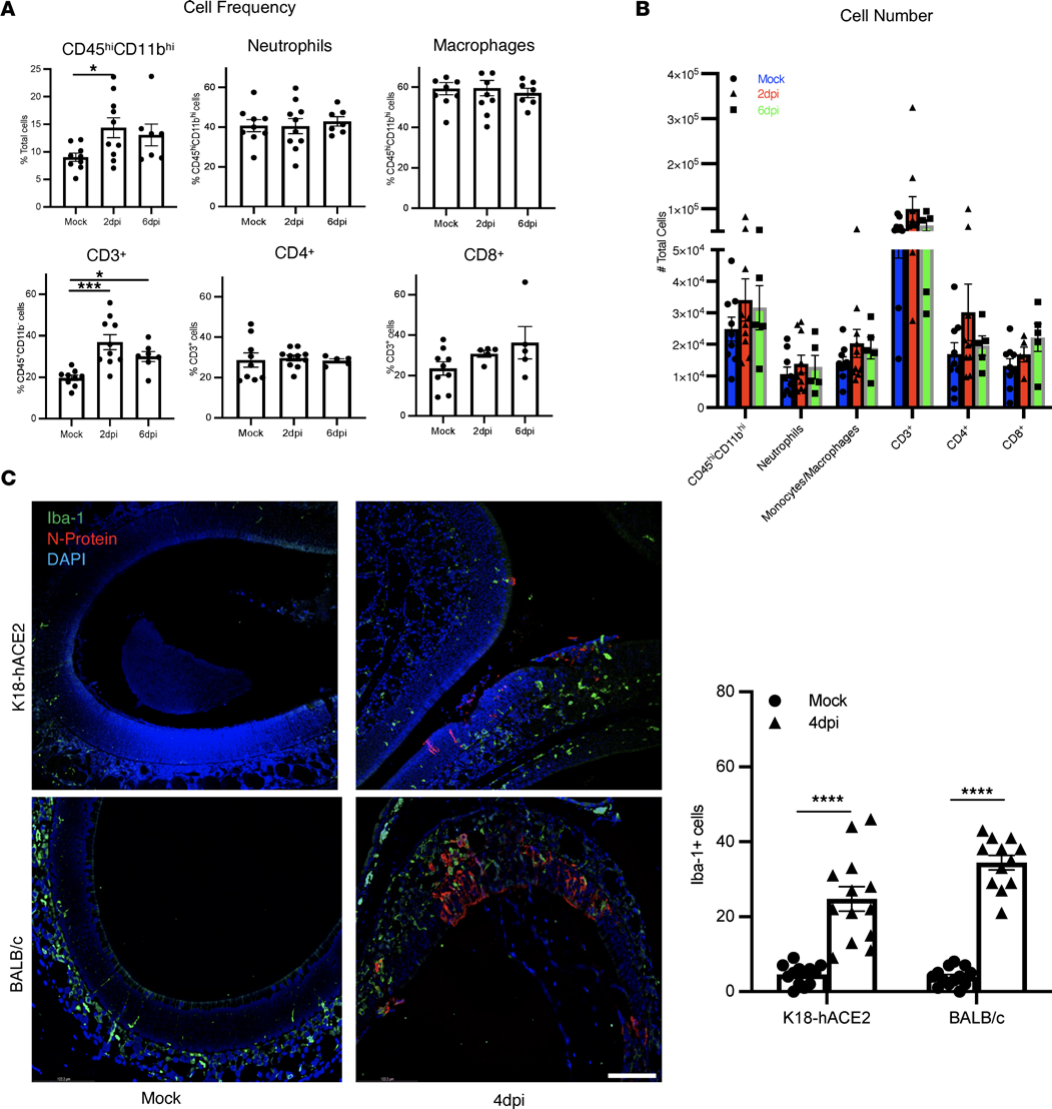
Inflammatory cell migration into OE after SARS-CoV-2 infection. (**A** and **B**) K18-hACE2 mice were infected with SARS-CoV-2. OE was harvested and analyzed for frequency (**A**) and numbers of indicated immune cells infiltrating the OE by flow cytometry (**B**). Data represent mean ± SEM of results pooled from 2 independent experiments with mock (8 mice), 2 dpi (8 mice), and 6 dpi (7 mice). (**C**) OE was harvested from mock-infected and infected K18-hACE2 (upper panels) and BALB/c (lower panels) mice at 4 dpi, and myeloid cells were stained with Iba1 (green). Three to 6 fields from 5–6 mice were analyzed. A representative set of sections is shown. Summary data represent Iba1^+^ cell numbers in the OE. Data represent mean ± SEM of results pooled from 2 independent experiments with 4 mice per group. Data were analyzed using Mann-Whitney *U* tests. ****P < 0.0001. Scale bar: 50 μm.

Inflammation has been reported to have a deleterious effect on the function of OSNs (49). To investigate the role of the virus-induced cellular inflammatory response in OSN damage, we analyzed the composition of cells infiltrating the OE using flow cytometry (gating shown in Supplemental Figure 3A). Flow cytometric analyses revealed an increased frequency of CD45^hi^CD11b^hi^ cells at 2 dpi, and CD3^+^ T cells at 2 and 6 dpi, but no difference in cell numbers in infected K18-hACE2 compared with mock-infected mice. We observed no differences in the number or frequency of monocytes/macrophages or neutrophils in CD45^hi^CD11b^hi^ cell populations, or in CD4^+^ and CD8^+^ T cells in CD3^+^ cell populations, in infected OEs at 2 dpi and 6 dpi (Figure 6, A and B). Similar results were observed in infected BALB/c mice, although the frequency and number of CD8 T cells was increased in the OE at 2 dpi and 6 dpi (Supplemental Figure 3, B and C). However, even in the absence of substantial changes in numbers of inflammatory cells, there were changes in myeloid cell distribution in olfactory tissue. Very few Iba1^+^ cells were detected in the uninfected OE, primarily in the lamina propria. However, after infection, the number of Iba1^+^ cells in the OE increased, and these cells were localized to cellular sites of infection (Figure 6C). Nearly identical results were found in SARS2-N501Y_MA30_–infected BALB/c mice (Figure 6C). These changes in myeloid cell distribution may have contributed to OSN dysfunction.

**Figure 7:**
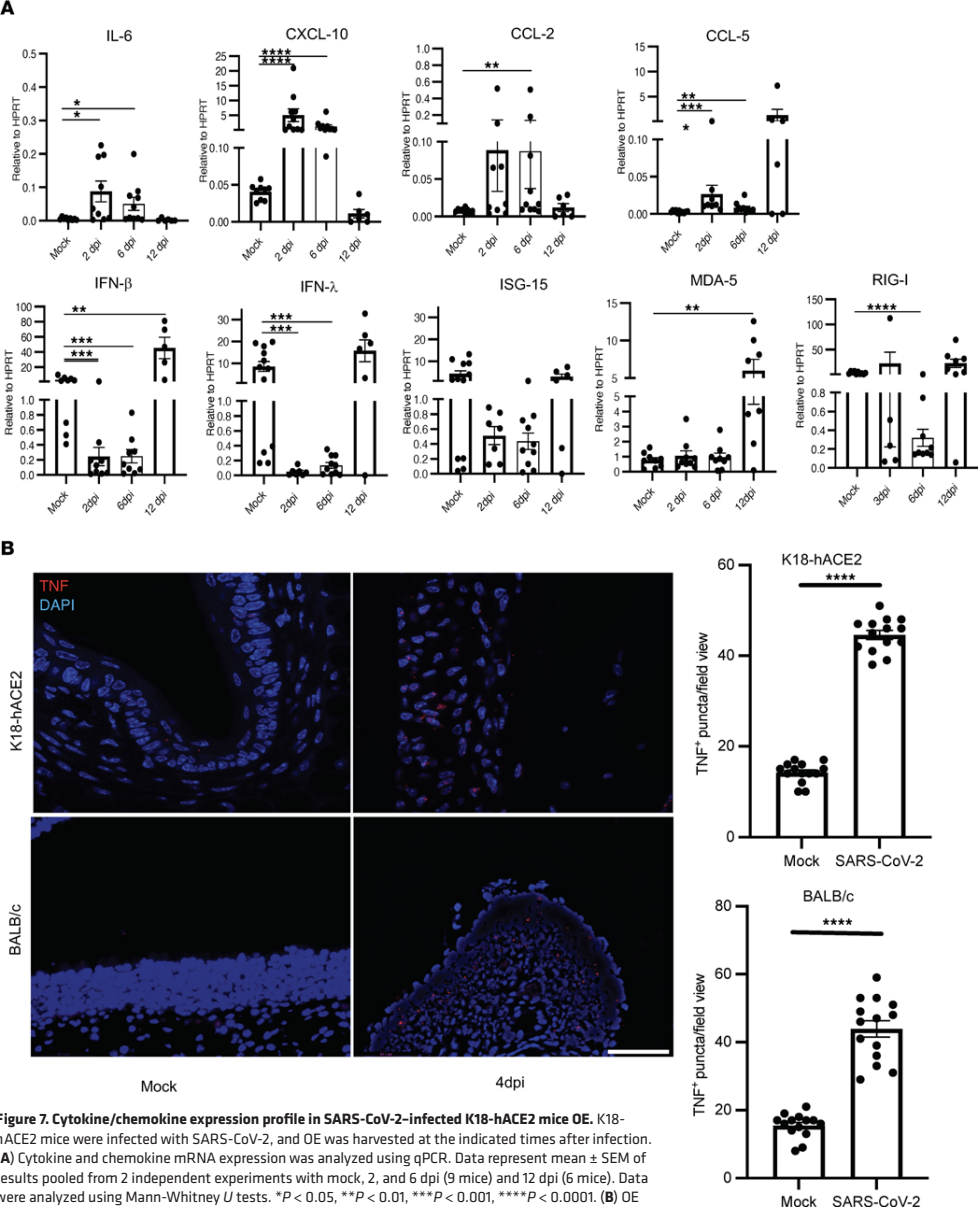
Cytokine/chemokine expression profile in SARS-CoV-2–infected K18-hACE2 mice OE. K18-hACE2 mice were infected with SARS-CoV-2, and OE was harvested at the indicated times after infection. (**A**) Cytokine and chemokine mRNA expression was analyzed using qPCR. Data represent mean ± SEM of results pooled from 2 independent experiments with mock, 2, and 6 dpi (9 mice) and 12 dpi (6 mice). Data were analyzed using Mann-Whitney *U* tests. *P < 0.05, **P < 0.01, ***P < 0.001, ****P < 0.0001. (**B**) OE were analyzed for TNF expression by RNAScope as described in Methods. Four mice and 4–5 sections per mouse were analyzed. Representative sections are shown. Scale bar: 50 μm.

Since myeloid cells are expected to be major cellular sources of proinflammatory cytokines and chemokines, which could contribute to OD, we examined cytokine/chemokine mRNA expression in the OE of infected K18-hACE2 and BALB/c mice. While proinflammatory cytokine and chemokine expression was changed as a result of SARS-CoV-2 infection, the patterns of expression differed in the 2 strains of mice. mRNA levels of genes encoding proinflammatory cytokines such as IL-6, CXCL10, CCL2, and CCL5 were significantly upregulated in infected K18-hACE2 mice at 2 and 6 dpi (Figure 7A). More notably, levels of IFN-I, IFN-III, and an IFN-stimulated gene, ISG15, were downregulated in infected compared with mock-infected OE in these mice. mRNA levels of all of these molecules normalized by 12 dpi, coincident with virus clearance from sustentacular cells. In contrast, IFN levels were unchanged in SARS2-N501Y_MA30_–infected BALB/c mice, while ISG15 levels increased at 6 dpi. (Supplemental Figure 2). mRNA levels of several proinflammatory cytokines and chemokines (MDA5, CCL2, RIG-I, and CXCL10) were generally decreased at 1 or more times after infection, but only differences in RIG-I and MDA were statistically significant.

Of note, we could not detect TNF mRNA in these assays, even when several different sets of primers were used. Since TNF is a major factor in SARS-CoV-2–induced inflammation (50), we further investigated TNF expression at the site of infection using RNAScope. Using specific probes, we observed increased numbers of TNF^+^ cells in the OE of infected as compared with control K18-hACE2 and BALB/c mice (Figure 7B). These results indicate that TNF was upregulated in both infected K18-hACE2 and BALB/c mice, which may contribute to OSN damage and subsequent OD.

### Sustentacular cell infection in mice infected with other coronaviruses.

**Figure 8:**
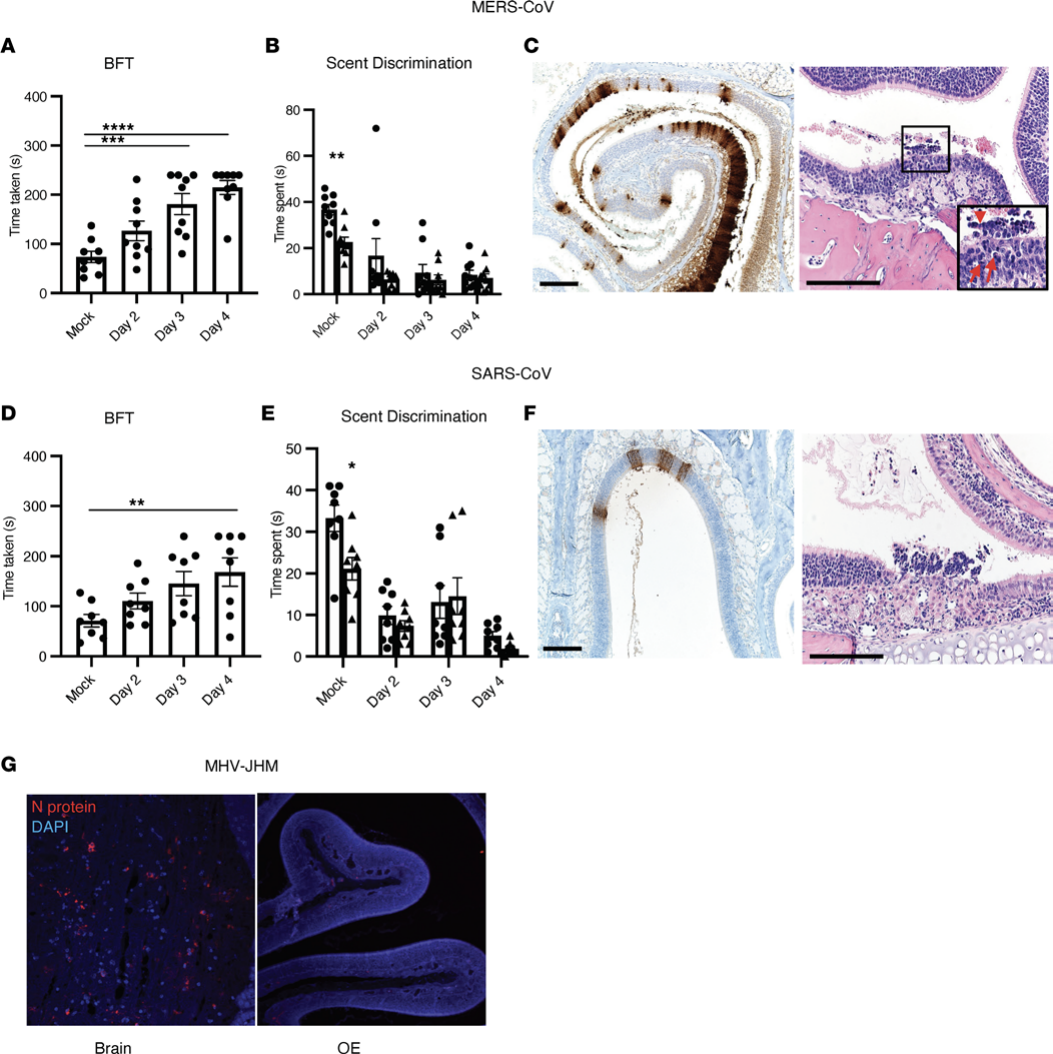
Assessment of sustentacular cell infection and anosmia in mice infected with MERS-CoV, SARS-CoV, or a murine CoV. (**A**–**C**) hDPP4-KI mice were infected with MERS-CoV and analyzed for anosmia/hyposmia by BFT and scent-discrimination tests (**A** and **B**). Data represent mean ± SEM of results pooled from 2 independent experiments with 9 mice. (**C**) Sections from MERS-CoV–infected OE were analyzed for viral antigen (left panel) and pathological changes (right panel). Pathological analysis shows damaged and disrupted OE after infection. In some regions, there were pyknotic and karyorrhectic nuclear debris (arrows) with cellular debris (arrowhead) sloughing in the lumen. Scale bar: 166 μm. (**D**–**F**) Fifteen-week-old B6 mice were infected with SARS-CoV and analyzed for anosmia/hyposmia by BFT and scent-discrimination tests (**D** and **E**). (**F**) Sections from SARS-CoV–infected OE were analyzed for viral antigen (left panel) and pathological changes (right panel). Scale bar: 166 μm. (**G**) Five- to 6-week-old B6 mice were infected intranasally with the neurovirulent JHM strain of MHV. N protein–positive (red) cells were identified in the brain but not in OE. (**A**, **B**, **D**, and **E**) Data represent the mean ± SEM of results pooled from 2 independent experiments with 8 mice per group. Data were analyzed by 1-way (**A** and **D**) and 2-way (**B** and **E**) ANOVA. *P < 0.05, **P < 0.01, ***P < 0.001, ****P < 0.0001. (**C**, **F**, and **G**) Representative sections from 4–6 individual mice are shown.

Anosmia and sustentacular cell infection have been reported in patients infected with SARS-CoV-2 but only rarely in the context of other human CoV infections (51). To assess the possibility that anosmia was present in patients with SARS or Middle East respiratory syndrome (MERS), both caused by highly pathogenic human CoV infections, we infected mice with MERS-CoV or SARS-CoV and examined them for sustentacular cell infection and OD. Since mice are naturally resistant to infection with MERS-CoV, we infected mice “knocked-in” for the MERS-CoV receptor, human dipeptidyl peptidase4 (hDPP4; hDPP4-KI mice) with mouse-adapted MERS-CoV (1 × 10^3^ plaque-forming units [pfu]) (52). Assessment of olfactory behavior and scent discrimination in MERS-CoV–infected mice showed that they were not able to find hidden food or distinguish novel from familiar dander at 2–4 dpi (Figure 8, A and B). Next, we collected OE from MERS-CoV–infected mice at 4 dpi. MERS N-protein antigen staining showed extensive infection of sustentacular cells (Figure 8C). Pathological analysis revealed disruption of the OE with pyknotic and karyorrhectic nuclear debris with cellular debris sloughing in the lumen (Figure 8C), similar to observations made in SARS-CoV-2–infected mice. Additionally, 12- to 15-week-old C57BL/6 mice were infected with mouse-adapted SARS-CoV (53) intranasally (1 × 10^4^ pfu) and assessed for hyposmia/anosmia on 2–4 dpi. Most mice were not able to find buried food by 3 and 4 dpi and were not able to discriminate between novel and familiar scents (Figure 8, D and E). SARS-CoV N protein staining of these sections revealed sustentacular cell infection (Figure 8F). We then analyzed the OE after intranasal infection with a murine coronavirus, the neurotropic JHM strain (1 × 10^4^ pfu) of mouse hepatitis virus (MHV-JHM) (54). While we were able to detect abundant viral antigen in the brain, no infected cells were identified in the nasal cavity (Figure 8G), demonstrating that sustentacular cell infection is not common to all CoV infections. SARS-CoV and MERS-CoV primarily infect the human lower respiratory tract (55–58), providing a possible explanation for why patients do not develop anosmia. Our results raise the possibility that anosmia would occur in infected patients if either of these viruses gained the capacity to infect the upper airway.

## Discussion

OD in SARS-CoV-2–infected patients is a characteristic symptom (9–12), but the underlying cause is not well elucidated. Here, we have shown that SARS-CoV-2 infection of the mouse OE leads to pathological changes at early times after infection. The damage that we observed likely contributes to OD. We found that tissue damage progressed over the first few days of the infection, with nearly full recovery by 15 dpi. Acute COVID-19–related anosmia generally has a favorable prognosis, with time to complete recovery generally varying between a few days to a few weeks in most patients (25–28). However, less is known about the long-term consequences of the persistent anosmia that occurs in some patients. Some COVID-19 survivors have long-term neurological and psychological sequelae (59, 60), and determining the relationship between these sequelae and loss of the senses of smell and taste will be critical (8). Long-term neurological sequelae and neurodegenerative disease were observed in patients who survived the 1918 influenza pandemic (61, 62). For example, encephalitica lethargica and Parkinson’s disease were well-described complications of the 1918 pandemic, causing substantial morbidity and mortality (63). It will be important to monitor COVID-19 survivors for similar complications (8).

Most studies, including this one, have shown that sustentacular cells are major sites of infection in patients (19) and experimentally infected animals, especially mice and hamsters (15–18). Whether OSNs are infected to a significant extent in patients remains controversial (17, 19, 64). Although sustentacular cells, but not OSNs, express ACE2 and TMPRSS2, some studies suggest that SARS-CoV-2 infects a small number of OSNs (16–18, 23, 65). The detection of viral products in OSNs may represent phagocytosis of infected cell debris (i.e., efferocytosis) rather than active infection, since neurons are known to ingest particulate matter (66, 67). In any case, our data indicate that OSNs were not optimally functional, since we observed fewer TH^+^ cells in OB. TH expression in the OB depends upon normal input from OSNs; thus, this finding is consistent with OSN dysfunction.

While the role of sustentacular cells is not fully known, they serve as supporting cells in the OE. They produce neurotrophic and neuromodulatory molecules such as endocannabinoids, insulin, and ATP (68–71). Sustentacular cells also phagocytose dead and dying cells and eliminate noxious substances (72). Internalization of odorant binding protein (OBP)/odorant complexes by sustentacular cells is critical for the rapid clearance of odorants and is required for continued responsiveness to odors (73). Most importantly, sustentacular cells also regulate the extracellular ionic environment required for normal functioning of the neurons (74–76). Sustentacular cells express metabotropic P2Y purinergic receptors and muscarinic acetylcholine receptors, which have roles in calcium signaling and in integrating communication between neurons, basal cells, and sustentacular cells (77–79). ATP, also produced by sustentacular cells, is important in neuroprotection and neuroproliferation and, thus, in proper functioning of OSNs (80–82). Together, these reports suggest that SARS-CoV-2 infection of sustentacular cells could have adverse effects on their physiological function, possibly explaining why widespread OD occurs after infection of a relatively small fraction of cells in the OE.

Cilia are critical components of the olfactory sensory pathway; our results demonstrate cilia loss and damage after SARS-CoV-2 infection. As mentioned above, the olfaction process begins with odorant binding to receptors on cilia (83–86). The loss and altered morphology of cilia almost certainly contributes to the OD observed in SARS-CoV-2–infected mice. Furthermore, our results indicated downregulation of genes encoding G_olf_, CNGA2, and ACIII — molecules critical for olfactory signal transduction as early as 2 dpi. We observed normalization of expression of these genes at 12 dpi, prior to structural recovery of cilia by 15 dpi. Full recovery of olfactory function, however, lagged behind that of cilia structure and of baseline levels of signaling mRNAs. Together, these results indicate that cilia that appeared to have intact structures at 15 dpi were probably not fully functional, but that function returned in the following days.

The presence of defective cilia and downregulated transduction molecules prompted us to examine OR expression. As described above, while OR mRNA levels were generally downregulated in both SARS-CoV-2–infected K18-hACE2 mice and SARS2-N501Y_MA30_–infected BALB/c mice, the patterns of expression differed. OR mRNA expression in infected K18-hACE2 mice revealed clear downregulation of all of the analyzed ORs at 2 dpi and 6 dpi, with full recovery by 12 dpi. In contrast, downregulation and subsequent recovery of OR mRNA expression in SARS2-N501Y_MA30_–infected BALB/c mice were variable. Remarkably, there was an inconsistent relationship between OR mRNA expression in BALB/c mice at early and late times after infection, when compared with mock-infected mice. Some OR mRNAs were not changed at early times after infection but exhibited increased expression compared with mock-infected samples at 22 or 40 dpi, while others decreased at early times and then returned to normal amounts during recovery. Every odor consists of several odorants, each of which is recognized by a single OR. Odors are recognized in a combinatorial fashion (87–89). The altered profile of OR mRNA expression during the recovery phase raises the possibility that a flawed combination of information is transmitted by OSNs, resulting in parosmia (distorted sense of smell) and phantosmia (olfactory hallucinations) in mice and, potentially, in COVID-19 survivors.

In other settings, OD has been reported to result from effects of local inflammation, often with associated OSN death (49). In 1 study, administration of LPS into a nare of a mouse was shown to cause ipsilateral neutrophil infiltration, cilia damage, and cell death. While we observed no changes in total inflammatory cell numbers after SARS-CoV-2 infection, we did detect a change in the distribution of monocytes/macrophages in the OE, consistent with migration from basal layers of the OE to positions proximate to infected cells in both BALB/c and K18-hACE2 mice. The extent of infiltration was greater in infected K18-hACE2 compared with BALB/c mice. This difference in the pattern of infiltration could contribute to differences in expression of proinflammatory molecules, since monocyte/macrophage expression of these molecules is well described in COVID-19 (90–93).

Notably, we observed increased expression of proinflammatory molecule mRNAs in the OE, with clear differences between infected K18-hACE2 and BALB/c mice. In infected K18-hACE2 mice, levels of several cytokines/chemokines increased, while those of IFN-I and associated ISGs decreased. SARS-CoV-2 has been shown previously to actively suppress IFN-I and ISG induction in infected cells (94–99), but systemic IFN-I expression is also delayed and diminished in patients with severe disease (100–103). Since IFN-I production and signaling should also occur in uninfected cells, the basis of this apparent inhibition of IFN-I production in bystander cells is not known. However, similar mechanisms may be occurring in the infected K18-hACE2 OE. In contrast, levels of IFN-β/λ did not change appreciably in infected BALB/c mice.

We also observed sustentacular cell infection and anosmia in mice infected with SARS-CoV and MERS-CoV. While SARS was occasionally associated with anosmia during the 2002–2004 epidemic (51), OD has not been reported in MERS. Many MERS patients are critically ill (55), so anosmia may be less likely to be detected. Of note, human SARS and MERS are characterized by extensive lower respiratory tract infection with less infection of the nasopharyngeal cavity (55, 58, 104), perhaps decreasing the likelihood of sustentacular cell infection. Even with these caveats, our results suggest that MERS survivors should be monitored for hyposmia and anosmia.

COVID-19–associated OD is a hallmark of SARS-CoV-2 infection. Olfactory impairment is believed to resolve within few weeks of onset, but due to the lack of long-term follow up, the exact proportion of with incomplete recovery is not known. Here, we showed that SARS-CoV-2 infection in mice results in tissue damage that leads to OD. We observed, as time progressed, that the infection resolved with a return to a normal sense of smell in most mice, yet hyposmia/anosmia persisted in a few. Our data demonstrate a loss of OR expression due to SARS-CoV-2 infection, despite a lack of neuronal infection, emphasizing the key roles that sustentacular cells have in OSN function. Further understanding of the role of sustentacular cells in olfaction, especially after viral infection, will help identify targets for therapeutic interventions in anosmia, whether induced by viruses or other environmental insults.

## Methods

### Animal and virus.

Twelve-week-old K18-hACE2 (The Jackson Laboratory) or BALB/c male and female mice (Charles River Laboratories) were used in all studies. K18-hACE2 mice, which contain 8 copies of the K18-hACE2 transgene, were infected intranasally with 2,000 pfu of the 2019n-CoV/USA-WA1/2019 strain of SARS-CoV-2 (accession no. MT985325.1) in 50 μL. The virus was passaged on Calu-3 2B4 cells (ATCC HTB-55). BALB/c mice were infected intranasally with SARS2-N501Y_MA30_ (1 × 10^3^ pfu), as previously described (37). Specific pathogen–free human DPP4–KI mice were generated as described previously and infected intranasally with 700 pfu mouse-adapted MERS-CoV infection in 50 μL (52). Twelve- to 16-week-old male and female mice were used for these studies. Five- to 6-week-old C57BL/6 mice were infected intranasally with 1 × 10^4^ to 4 × 10^4^ pfu of MHV-JHM in 10 μL (54). C57BL/6 mice were purchased from Charles River Laboratories for SARS-CoV and MHV studies. Mouse-adapted SARS-CoV (MA15) was a gift from Kanta Subbarao (NIH).

### Olfactory behavior analysis.

Olfactory behavior evaluation was performed using BFTs and social scent-discrimination tests, as described previously (15). Briefly, in BFTs, mice were sensitized with Froot Loops (Kellogg Cereals) for a week before infection. During testing, mice were allowed to find the food hidden under the bedding, and the time required to find food was recorded. If mice did not find food within 240 seconds, the time was marked as 240 seconds. For social scent-discrimination testing, mice were allowed to explore tubes containing dander from an unfamiliar (“novel”) or a familiar cage for 3 minutes. Time spent on each tube was recorded and compared on the indicated days after infection.

### Histology and IHC.

Mice were anesthetized by i.p. injection of ketamine-xylazine and perfused transcardially with PBS. Tissues were fixed in zinc formalin. For routine histology, tissue sections (4 μm each) were stained with H&E.

### Confocal imaging.

For immunofluorescence assays, OE were fixed in zinc formalin, decalcified using EDTA, and embedded in paraffin. Sections were deparaffinized and processed for citrate-based antigen retrieval (Vector Laboratories) according to the manufacturer’s protocol. Sections were washed 3 times for 5 minutes in PBS before treatment with 0.1% Triton X-100 in PBS for 20 minutes. Sections were then rinsed in PBS followed by incubation with CAS block (Invitrogen, Thermo Fisher Scientific) for 60 minutes. Primary antibodies against Iba1 (Wako, 1:1,000), SARS-CoV-2 N protein (Sino Biological, 1:5,000), SARS-CoV-2–N protein, OMP (Abcam, 1:1,000), Ki-67 (Cell Signaling, 1:1,000), MERS-CoV–N protein (Sino Biological, 1:1,000), Ac–β-tubulin (Cell Signaling, 1:1,000), and TH (Novus, 1:1,000) were used. Sections were rinsed before incubation with a 1:1,000 dilution of an appropriate Alexa Fluor 546–conjugated (A546-conjugated, catalog A11018) or A488-conjugated (catalog A11070) goat anti-mouse or anti-rabbit antibody (Thermo Fisher Scientific). After a final wash with PBS, slides were mounted with Vectashield antifade reagent containing DAPI (Vector Laboratories). Images were obtained using a Carl Zeiss 710 confocal microscope. Three different areas were imaged for every brain section for cell counting. ImageJ (NIH) was used for image processing and cell counting. Zeiss 710 and Leica microscopes were used to capture images.

### RNA extraction, PCR, and primers.

K18-hACE2 and BALB/c mice were deeply anesthetized with ketamine/xylazine and perfused transcardially with PBS. The nasal cavity was visualized and the OE isolated into Trizol (Thermo Fisher Scientific). RNA was extracted from OE per manufacturer’s protocol. mRNA expression levels were analyzed by quantitative PCR (qPCR). The primer sets used for PCR are listed in Table 1. SARS-CoV-2–N primer was purchased from IDT (catalog 10007032). The expression levels were normalized to expression of hypoxanthine guanine phosphoribosyltransferase (HPRT) or GAPDH by the following CT equation: ΔCT = CT of the gene of interest – CT of HPRT or GAPDH. All results are shown as a ratio to HPRT or GAPDH calculated as 2^−ΔCt^.

### Antibodies and flow cytometry.

K18-hACE2 or BALB/c mice were deeply anesthetized with ketamine/xylazine OE isolated as described above. Isolated OE was digested with 1 mg/mL collagenase D (Roche) and 0.1 mg/mL DNase I (Roche) at 37°C for 20 minutes. Flow cytometric staining was performed on OE for immune cell analysis using the following antibodies: CD3-PE (clone 145-2C11; BioLegend), CD11b-eFluor 450 (clone M1/70; eBioscience), CD45-phycoerythrin-Cy7 (CD45-PE-Cy7) (clone 30-F11; BioLegend), CD4-PerCP-Cy5.5 (clone RM4-5; BioLegend), CD8-APC-Cy7 (clone 53–6.7; eBioscience), and Ly-6G-APC (neutrophil marker) (BioLegend). Cells were treated with anti-CD16/32, clone 2.4G2 generated in-house, to block nonspecific Fc receptor binding and were stained with the indicated antibodies at 4°C. Data were acquired with a BD FACSVerse cytometer and analyzed using FlowJo software (Tree Star Inc.).

### Scanning electron microscopy.

For scanning electron microscopy, mice were perfused intracardially with PBS. Mouse OE were fixed in 2.5% glutaraldehyde in 0.1M sodium cacodylate buffer overnight at 4°C and rinsed twice with 4% paraformaldehyde solution. Samples were rinsed with 0.1M sodium cacodylate buffer 3 times for 20 minutes to remove residual fixative before osmication and dehydration. Samples were gently submerged in a solution of 1% OsO_4_ in deionized water for 90 minutes. Samples were washed for 20 minutes in 0.1M sodium cacodylate buffer. After dehydration using graded concentrations of ethanol, samples were critical-point dried, mounted on a stub, and analyzed by field-emission scanning electron microscopy with a Hitachi S4800 scanning electron microscope.

### RNAScope.

RNAScope was performed according to the manufacturer’s protocol (Advanced Cell Diagnostics [ACD]). Fixed frozen paraffin-embedded OE sections were deparaffinized in xylene and 100% ethanol and dried. Target retrieval was performed (RNAScope Target Retrieval Reagents, ACD, 322000) after hydrogen peroxide treatment and was followed by protease treatment (RNAScope H_2_O_2_ and Protease Plus, ACD, 322330). Probes for RNAs of SARS-CoV-2 S-Protein (RNAScope Probe V-nCoV-2-S-C2) and TNF (RNAScope Probe Mm-TNFa-C3) were mixed and incubated on slides for in situ hybridization. Following signal detection (ACD Kit), slides were stained with DAPI and mounted with DAPI Mounting Media (ACD).

### Statistics.

Buried-food data were analyzed using 1-way ANOVA. Scent-discrimination tests were analyzed using 2-way ANOVA. PCR and cell counting data were analyzed using a Mann-Whitney *U* test. *P* < 0.05 was considered significant. Data in graphs are presented as mean ± SEM.

### Study approval.

All animal studies were approved by the University of Iowa IACUC and met the stipulations of the *Guide for the Care and Use of Laboratory Animals* (National Academies Press, 2011).

## Author contributions

Study design was contributed by SP and AKV. Experiments were conducted by AKV, JZ. AKV, DKM, and SP acquired and analyzed data. Manuscript preparation was contributed by AKV, DKM, and SP.

## Supplementary Material

Supplemental data

## Figures and Tables

**Table 1:**
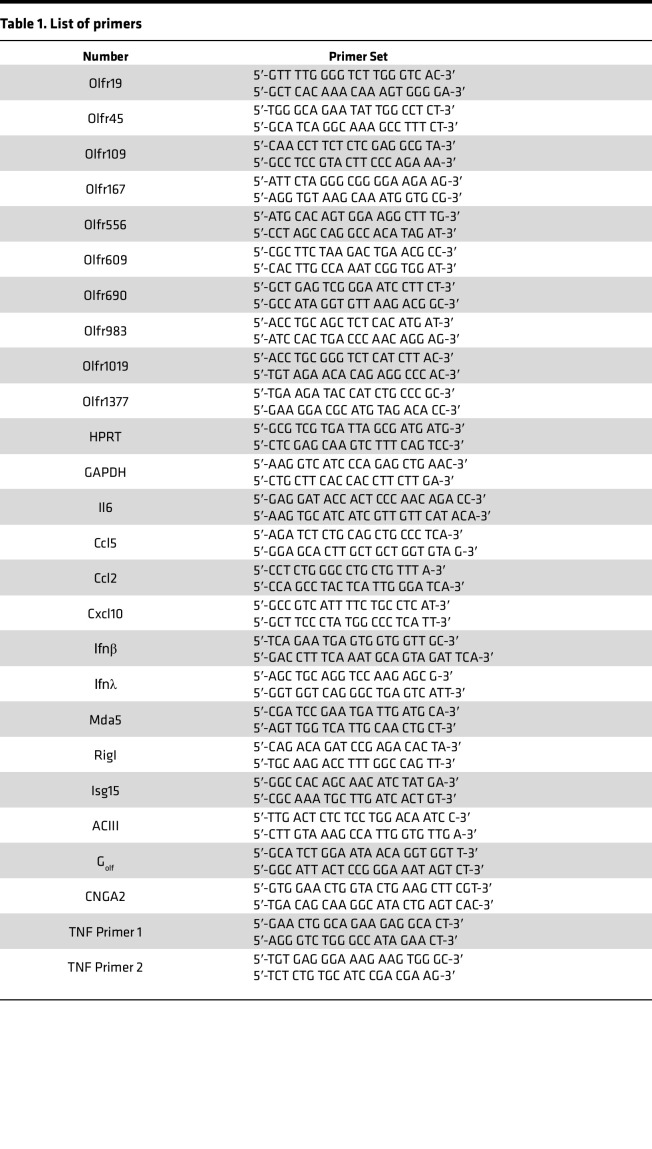
List of primers

## Version 1 11/15/2022

In-Press Preview

## Version 2 12/22/2022

Electronic publication
