# Supplementary material for: SARS-CoV-2 infection of sustentacular cells disrupts olfactory signaling pathways
Source: JCI Insight. 2022 Dec 22;7(24):e160277. doi: 10.1172/jci.insight.160277 (PMC9869979; doi:10.1172/jci.insight.160277)
Supplement: Supplemental data [file jciinsight-7-160277-s109.pdf]

SARS-CoV-2 infection of sustentacular cells disrupts olfactory signaling pathways

Abhishek Kumar Verma[1], Jian Zheng[1], David K. Meyerholz[2], Stanley Perlman[1#]

[1] Department of Microbiology and Immunology, University of Iowa, Iowa City, IA, USA

[2] Department of Pathology, University of Iowa, Iowa City, IA, USA

[#]Corresponding Author. Stanley Perlman, Department of Microbiology and Immunology, University of Iowa, Iowa City, IA 52242, tele 319-335-8549, email: Stanley-perlman@uiowa.edu

Keywords: SARS-CoV-2, Anosmia, COVID-19, Odorant Receptors, Cilia

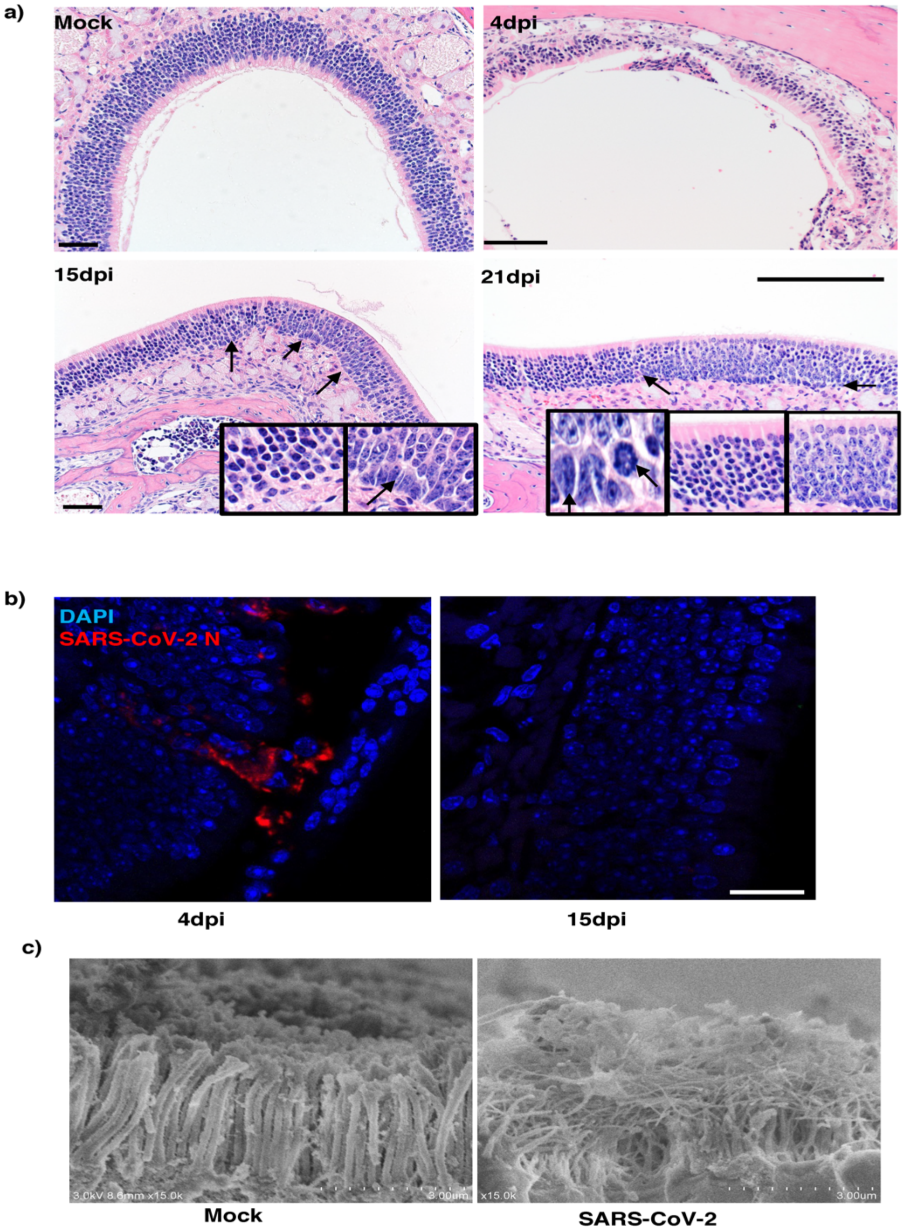

Fig S1

Supplementary Figure 1. Detection of viral RNA, and pathological changes in infected mice. (a) Histoopathological analysis of OE from SARS2-N501Y$_{MA30}$-infected BALB/c mice shows disruption and recovery of OE. Uncommon sites of basal cell hyperplasia (arrow 15dpi) and hyperchromatic OSN nuclei were observed at 21 dpi (left inset, arrow; middle inset). Occasional fields of OE that were composed mostly of regenerative epithelia with larger nuclei (right inset,

arrows) were observed. Scale bar represents 83μm (b) Sections were prepared at the indicated times p.i. and analyzed by RNAScope for viral RNA (red). Scale bar 50μm. (c) SEM analysis shows uniform lawn of normal-appearing cilia in mock-infected OE but damaged cilia at 4dpi in K-18-hACE2 mice. Scale bar 3μm.

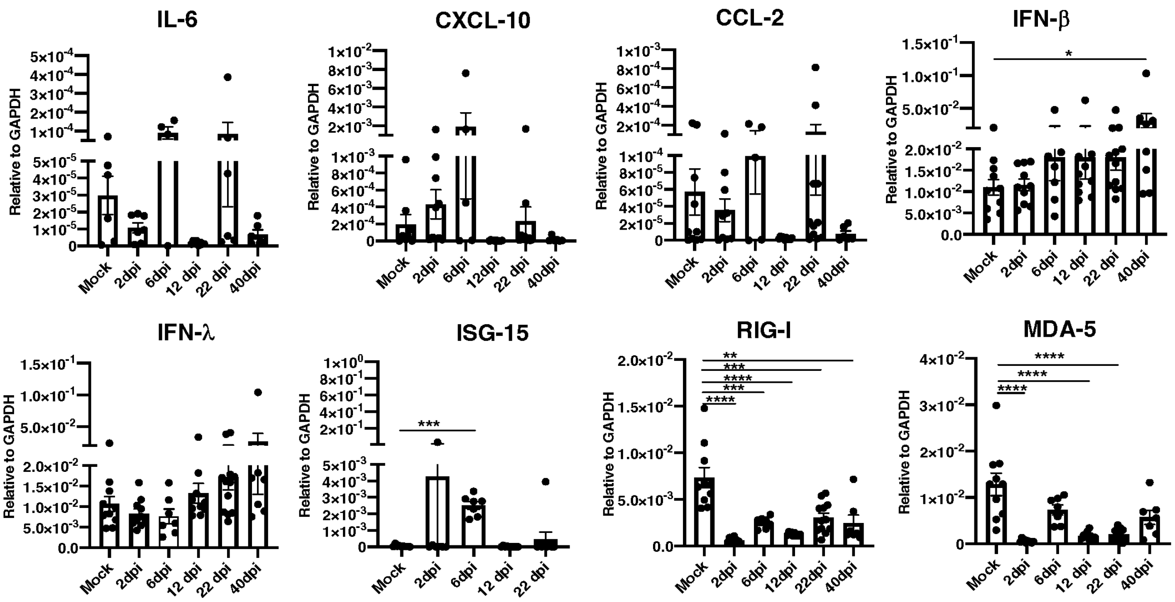
IL-6
CXCL-10
CCL-2
IFN-β
IFN-λ
ISG-15
RIG-I
MDA-5
Relative to GAPDH
Mock
2dpi
6dpi
12 dpi
12dpi
22 dpi
22dpi
40dpi
*
***
**
****

Fig S2

Supplementary Figure 2. **Cytokine/chemokine expression profile in SARS-CoV-2 infected BALB/c mouse OE**. (a) SARS2-N501Y$_{MA30}$-infected BALB/c mice OE were harvested at the indicated times p.i. Cytokine and chemokine mRNA expression was analyzed using qPCR. Data represent means ± SEM of results pooled from two independent experiments with 7 mice per group. Data were analyzed using Mann-Whitney *U* tests. *P < 0.05, **P < 0.01, ***P < 0.001, ****P < 0.0001.

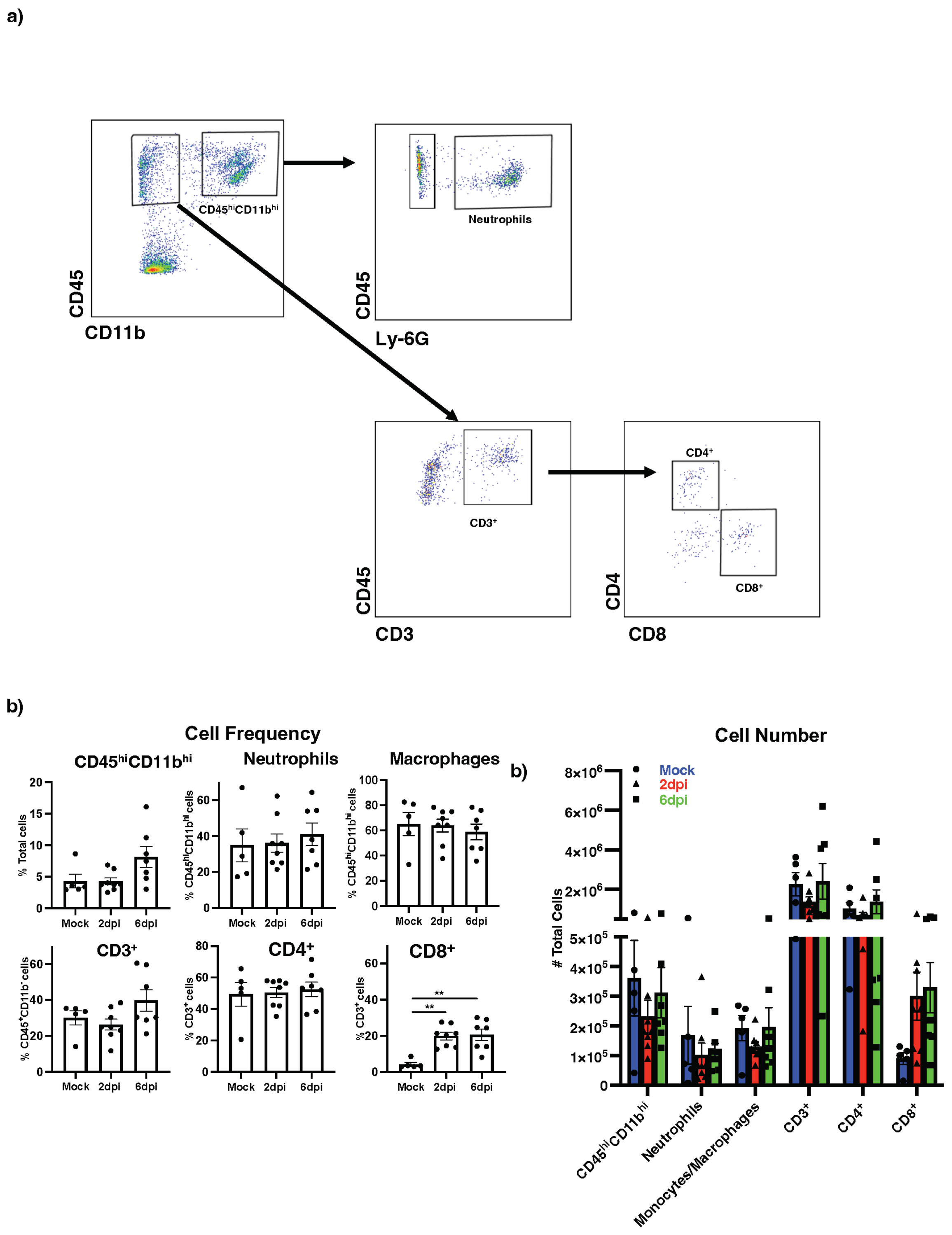
a)
CD45
CD11b
CD45hiCD11bhi
CD45
Ly-6G
Neutrophils
CD45
CD3
CD3+
CD4
CD8
CD4+
CD8+
b)
Cell Frequency
CD45hiCD11bhi
Neutrophils
Macrophages
% Total cells
% CD45hiCD11bhi cells
% CD45hiCD11bhi cells
Mock
2dpi
6dpi
CD3+
CD4+
CD8+
% CD45+CD11b- cells
% CD3+ cells
% CD3+ cells
**
**
b)
Cell Number
Mock
2dpi
6dpi
Total Cells
8×10^6
6×10^6
4×10^6
2×10^6
5×10^5
4×10^5
3×10^5
2×10^5
1×10^5
0
CD45hiCD11bhi
Neutrophils
Monocytes/Macrophages
CD3+
CD4+
CD8+

Fig S3

Supplementary Figure 3. Immune cell infiltration in olfactory epithelium after SARS-CoV-2 infection. BALB/c mice were infected with SARS-CoV-2. (a) Gating strategy for flow cytometric analysis of cells from OE. OE was hcarvested and analyzed for frequency (b) and numbers of infiltrating immune cells by flow cytometry (c). Data represent means ± SEM of results pooled from two independent experiments with mock-5, 2 and 6 dpi-7 mice per group.
